# PDGF-BB/EGR1 Axis Drives Fibroblast Activation Protein Expression to Promote Abdominal Aortic Aneurysm

**DOI:** 10.7150/ijms.114429

**Published:** 2025-06-05

**Authors:** Zhihao Zhou, Lin Huang, Hui Luo, Rongzhou He, Ridong Wu, Rui Wang, Kangjie Wang, Chen Yao

**Affiliations:** 1 Division of Vascular Surgery, the First Affiliated Hospital, Sun Yat-sen University, Guangzhou 510800, China; National-Guangdong Joint Engineering Laboratory for Diagnosis and Treatment of Vascular Disease, First Affiliated Hospital, Sun Yat-sen University, Guangzhou 510080, China.; 2 Department of Interventional Medicine, The Fifth Affiliated Hospital, Sun Yat-sen University, Zhuhai 519000, China.; 3 Department of Nuclear Medicine, the First Affiliated Hospital, Sun Yat-sen University, Guangzhou 510800, China.

**Keywords:** Abdominal aortic aneurysm, fibroblast activation protein, vascular smooth muscle cells.

## Abstract

This study investigates the molecular mechanisms of fibroblast activation protein (FAP) in vascular smooth muscle cells (VSMCs) during abdominal aortic aneurysm (AAA) development. Bulk and single-cell RNA sequencing analysis revealed elevated FAP expression in AAA-derived VSMCs. In a porcine pancreatic elastase (PPE)-induced AAA mouse model, pharmacological inhibition of FAP (Ac-Gly-BoroPro) attenuated aneurysm formation and reduced macrophage infiltration. Further analysis showed that PDGF-BB upregulates FAP expression in VSMCs via the transcription factor EGR1, which binds to the FAP promoter to drive transcription. EGR1 inhibition significantly reduced PDGF-BB-induced FAP expression, highlighting its regulatory role. Additionally, clinical ^18^F-FAP inhibitor PET/CT imaging in an infectious AAA patient revealed strong FAP expression in the aneurysm wall. These findings underscore the importance of the PDGF-BB/EGR1/FAP axis in AAA pathogenesis and suggest that targeting FAP could offer therapeutic potential for managing AAA progression.

## Introduction

Abdominal Aortic Aneurysm (AAA) is defined by the localized expansion of the abdominal aorta, typically identified through imaging when the aortic diameter exceeds 30 mm 1. Currently, no pharmacological treatments effectively address aneurysms, leaving surgical and endovascular interventions as the sole therapeutic options 2. Consequently, discovering drugs capable of mitigating AAA formation and progression has been a longstanding focus of our research.

Fibroblast Activation Protein (FAP), a glycoprotein anchored to the cell membrane, consists of 760 amino acids. It includes a brief intracellular segment (six amino acids), a transmembrane portion (20 amino acids), and an extensive extracellular region (734 amino acids) 3. Belonging to the dipeptidyl peptidase family, FAP plays a role in various biological processes and shares up to 48% sequence homology with dipeptidyl peptidase IV (CD26). FAP exhibits both postproline peptidase and endopeptidase activities, enabling it to degrade extracellular proteins and facilitate tissue remodeling 4. Vascular smooth muscle cells (VSMCs), the primary cellular component of arterial walls, play critical roles in vascular contraction, proliferation, phenotypic modulation, and extracellular matrix (ECM) regeneration. In AAA, VSMCs contribute to disease progression through genetic alterations, ECM production and degradation, inflammatory responses, oxidative stress, and apoptosis 5. FAP was found to be expressed predominantly on VSMCs in atherosclerosis, deep vein thrombosis, and thoracic aortic aneurysms 6-8. However, the expression pattern and function of FAP in AAA have not been clarified, which requires validation.

Single-cell RNA sequencing (scRNA-seq) has emerged as a powerful tool for studying disease mechanisms. Unlike traditional RNA sequencing, scRNA-seq offers unparalleled resolution in analyzing cellular heterogeneity, enabling the identification of rare cell populations and prediction of intercellular signaling pathways 9. Recent studies have applied scRNA-seq to AAA research, highlighting its potential for uncovering novel insights into disease mechanisms and therapeutic targets 10. In this research, single-cell RNA sequencing (scRNA-seq) was utilized to explore the expression patterns of FAP. We also investigated the role and underlying mechanism of FAP in a murine model of porcine pancreatic elastase (PPE)-induced AAA and *in vivo* experiments. Additionally, we employed ^18^F-FAP inhibitor (FAPi) PET/CT imaging to assess FAP expression in a patient with infectious AAA, as the role of FAP, and FAPi-based PET imaging in cardiovascular disease has been increasingly recognized and may improve the assessment and treatment of patients with cardiovascular diseases 11.

## Methods

### Human sample collection

Tissue samples from AAA patients undergoing surgical repair were collected for analysis, with ethical approval (Authorization No. [2022] 939) granted by the First Affiliated Hospital of Sun Yat-sen University. Normal aortic tissues were collected from healthy donors for comparative analysis. All samples were processed for downstream experiments.

### Data acquirement

Six RNA datasets (GSE47472, GSE57691, GSE98728, GSE7084, GSE183464, GSE232911) and three scRNA-seq datasets (GSE166676, GSE237230, and GSE226492) were downloaded from the Gene Expression Omnibus (GEO) database for further investigation. Finally, GEO database GSE241545 was also retrieved to analyse differentially expressed genes (DEGs) of VSMCs treated with or without PDGF-BB.

### Bulk data preprocessing and analysis

To address batch effects and integrate multiple datasets for robust differential gene expression analysis, we employed a combination of computational approaches. For the microarray datasets (GSE47472, GSE57691, and GSE98728), batch effects were corrected using the ComBat method, and DEGs were identified using the “limma” R package. Additionally, Rank-In, which enables the integration of different microarray data by compensating for batch effects, was applied to further identify DEGs. For the RNA-seq datasets (GSE7084 and GSE183464), we also used the Rank-In method with a false discovery rate (FDR) < 0.05 were considered statistically significant DEGs. For the GSE241545 and GSE232911 dataset, differences in gene expression were systematically analyzed using the “limma” R package for microarray data. Furthermore, differences between normal and FAP-treated macrophages in GSE206637 were investigated using the “limma” R package. Support vector machine (SVM) and LASSO regression analysis were performed to screen candidate genes.

### scRNA-seq data preprocessing and analysis

Single-cell RNA datasets were analyzed using Seurat and FastMnn R packages. Cells expressing 200-5,000 genes were retained, and those with mitochondrial gene expression exceeding 30% were excluded. Principal component analysis (PCA) was performed using 12 principal components (PCs). Cell clusters were visualized via uniform manifold approximation and projection (UMAP) plots and annotated using the “FindAllMarkers” function, identifying the top 10 differentially expressed genes per cluster. Specific markers were used to classify cell types.

### Immune infiltration analysis

CIBERSORT was employed to estimate immune cell proportions in high- and low-FAP expression groups, with results visualized using box plots.

## Materials

Recombinant mouse PDGF-BB was purchased from Medchemexpress (HY-P7087, respectively, MCE). The primary antibodies for FAP (bs-5758R, Bioss, ab207178, abcam), EGR1 (4154, Cell Signaling Technology), ACTA2(67735, Proteintech), F4/80 (28463, Proteintech), CD68 (1:500, 76437, Cell Signaling Technology), and GAPDH (60004, Proteintech), and secondary anti-rabbit IgG HRP-linked antibody (SA00001-2, Proteintech) were also purchased.

### VSMCs culture and treatment

Mouse VSMCs (MOVAS cells, ATCC) were cultured in high-glucose DMEM supplemented with 10% fetal bovine serum and 1% penicillin/streptomycin at 37 °C in a 5% CO2 atmosphere. Cells were seeded at 2 × 10^5 cells/mL, allowed to adhere for 24 h, and treated with PDGF-BB (0-20 ng/mL) or saline for 24 h before analysis.

### siRNA transfection

VSMCs were transfected with EGR1 siRNA using Lipo3000 Transfection Reagent (ThermoFisher), adhering to the manufacturer's guidelines. Gene expression was assessed 48 hours post-transfection. The siRNAs were sourced from QINKE (Carlsbad, CA, USA), with the target sequence for mouse si-EGR1-1 being CAUGAACGCCCAUAUGCUU.

### Protein extraction and western blot

Proteins extracted from VSMCs were separated on 10-15% SDS-PAGE gels, transferred to nitrocellulose membranes, and incubated with primary and secondary antibodies. Protein bands were detected using chemiluminescence.

### RNA isolation and RT-qPCR

Total RNA was extracted using TRIzol, converted to cDNA, and quantified via RT-qPCR with SYBR Green (Accurate Biology, AG11701). GAPDH was used as the reference gene. Primer sequences are provided in Table 1.

### Animal studies

Male C57BL/6 mice (8-9 weeks old) were obtained from the First Affiliated Hospital of Sun Yat-sen University with authorization from the Institutional Animal Care and Use Committee (permit number: SYSU-IACUC-2022-001803). AAA was induced by PPE treatment for 10 min, followed by daily intraperitoneal injections of the FAP inhibitor (FAPi) Ac-Gly-BoroPro (10 mg/kg) 12 or saline. Aortic diameter was measured, and tissues were harvested 14 days post-surgery. AAA incidence was defined as a ≥50% increase in aortic diameter compared to controls.

### ChIP-qPCR analysis

ChIP-qPCR was conducted using the ChIP-IT High Sensitivity Kit (53040, Active Motif). Briefly, VSMCs were harvested and subjected to cross-linking using 1% formaldehyde for 15 minutes at ambient temperature. The reaction was halted by treating the cells with glycine for 5 minutes, followed by two PBS washes. Immunoprecipitation was performed using 10 μM anti-EGR1 antibody and 2 μg IgG antibody, adhering to the supplier's protocol (P2078, Beyotime, China). Subsequent qRT-PCR was conducted using FAP-specific primers (forward: 5′-GCCTGTCGCCTGCTCTAC-3′, reverse: 5′-CCAGCTTCCCTCTTTCTTCC-3′).

### Histological examination

Abdominal aortas from both murine and human sources were flushed with saline and preserved in 4% paraformaldehyde. These tissues were then paraffin-embedded and sliced into serial sections. Sections of 3-4 µm thickness were deparaffinized and stained with hematoxylin and eosin (HE) for morphological assessment, while Elastin Van Gieson (EVG) staining was employed to evaluate elastin degradation and collagen content. Elastin degradation was quantified by counting breaks per vessel.

As described previousl**y** 13, AAA and normal tissue samples were sectioned into 5 μm slices for immunohistochemistry (IHC) and immunofluorescence (IF). For IHC, tissue sections were incubated overnight at 4 °C with antibodies against FAP. For IF, sections were treated with primary antibodies against ACTA2 and FAP overnight at 4 °C. Slides were mounted and analyzed using confocal microscopy (Leica, Germany).

### PET imaging procedure and evaluation

This study was approved by the Ethics Committee of the First Affiliated Hospital of Sun Yat-sen University (Ethics Approval Number: [2024] 336). Patients with AAA underwent both ^18^F-FAPi-42 imaging, based on fibroblast activation protein (FAP) inhibitors, and ^18^F-FDG PET/CT scans, based on glucose metabolism, in the Department of Nuclear Medicine, as previously described 14. PET images were reconstructed using the line-of-response RAMLA algorithm, with the two scans performed at least 24 hours apart. Two nuclear medicine specialists independently evaluated the images, analyzing lesion location, maximum standardized uptake value (SUVmax), and target-to-background ratio (TBR) through semi-quantitative analysis.

### Statistical methods

Continuous data are expressed as mean ± standard deviation (SD), while categorical variables are presented as frequencies (percentages). The Shapiro-Wilk test assessed data normality, and the Levene test evaluated variance homogeneity. For two-group comparisons, Student's t-test (equal variances) or Welch's t-test (unequal variances) was applied for normally distributed data; otherwise, the Mann-Whitney U test was used. For multiple groups, two-way ANOVA with Bonferroni correction was employed. Categorical variables were compared using the Chi-squared test or Fisher's exact test. Analyses were conducted using the Xiantao tool (https://www.xiantao.love/) and GraphPad Prism 9.0, with a P-value < 0.05 considered statistically significant.

## Results

### Bulk data analysis of aortic cells from patients with AAA

**Figure 1:**
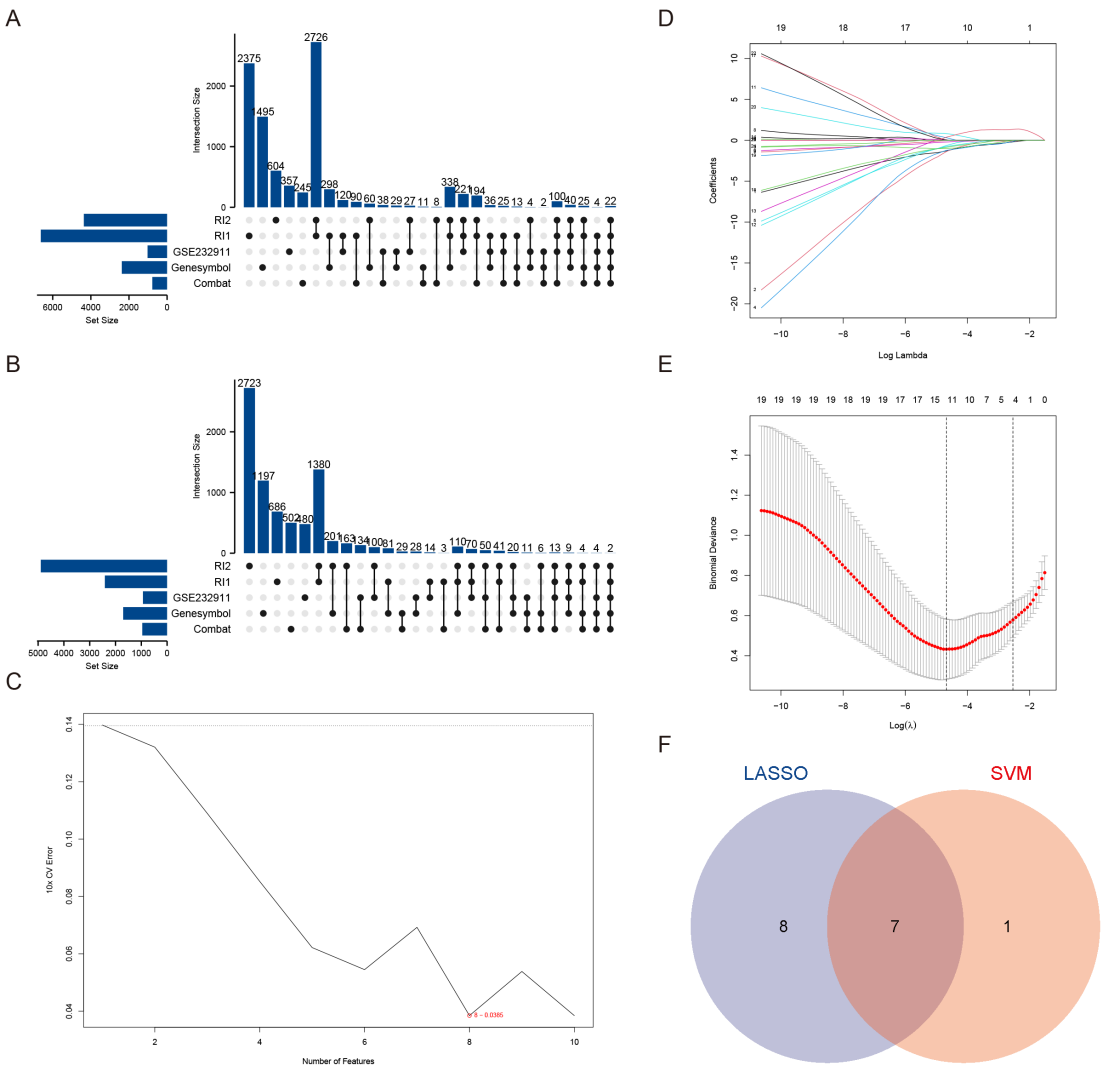
** Identification of candidate genes in AAA.** (A) Upset plot depicting the 22 commonly DEGs identified from bulk RNA sequencing data. RI1 represents DEGs obtained using the Rank-in method from the GSE47472, GSE57691, and GSE98728 datasets. RI2 represents DEGs obtained using the Rank-in method from the GSE7084 and GSE183464 datasets. (B) Upset plot illustrating the 2 commonly downregulated DEGs identified from bulk RNA sequencing data. RI1 represents DEGs obtained using the Rank-in method from the GSE47472, GSE57691, and GSE98728 datasets. RI2 represents DEGs obtained using the Rank-in method from the GSE7084 and GSE183464 datasets. (C) Characteristic genes selection via SVM-RFE algorithm. (D) LASSO coefficient profiles of the 43 genes. (E) Characteristic genes selection via LASSO algorithm. (F) Venn diagram of the intersection of characteristic genes from SVM and LASSO genes.

To systematically identify candidate genes associated with VSMCs in aortic aneurysm pathogenesis, we employed a multi-step bioinformatics approach. Our initial screening focused on differentially expressed markers specific to VSMCs 15, which served as the foundation for identifying critical molecular players. Through comparative analysis of six independent datasets, we detected 24 consistently dysregulated genes, comprising 22 upregulated and 2 downregulated genes (Figure 1A-B). More details were shown in Table 2.

Subsequent machine learning-based refinement using both SVM feature selection (maximum accuracy = 0.824, minimum RMSE = 0.0386) (Figure 1C) and Lasso regression analysis (Figure 1D-E) yielded seven high-confidence candidate genes via Venn diagram intersection (Figure 1F). These included: Fibroblast Activation Protein (FAP), FBJ murine osteosarcoma viral oncogene homolog B (FOSB), Ras association domain family member 2 (RASSF2), CKLF-like MARVEL transmembrane domain containing 7 (CMTM7), Early growth response 2 (EGR2), Tissue inhibitor of metalloproteinases 3 (TIMP3), and Interferon regulatory factor 8 (IRF8). Through integrated analysis of six datasets, we identified for the first time that both FAP and CMTM7 were among the most significantly upregulated genes in AAA, suggesting their potential central roles in AAA pathogenesis.

### ScRNA-seq analysis reveals cell-type specific expression patterns of candidate genes

**Figure 2:**
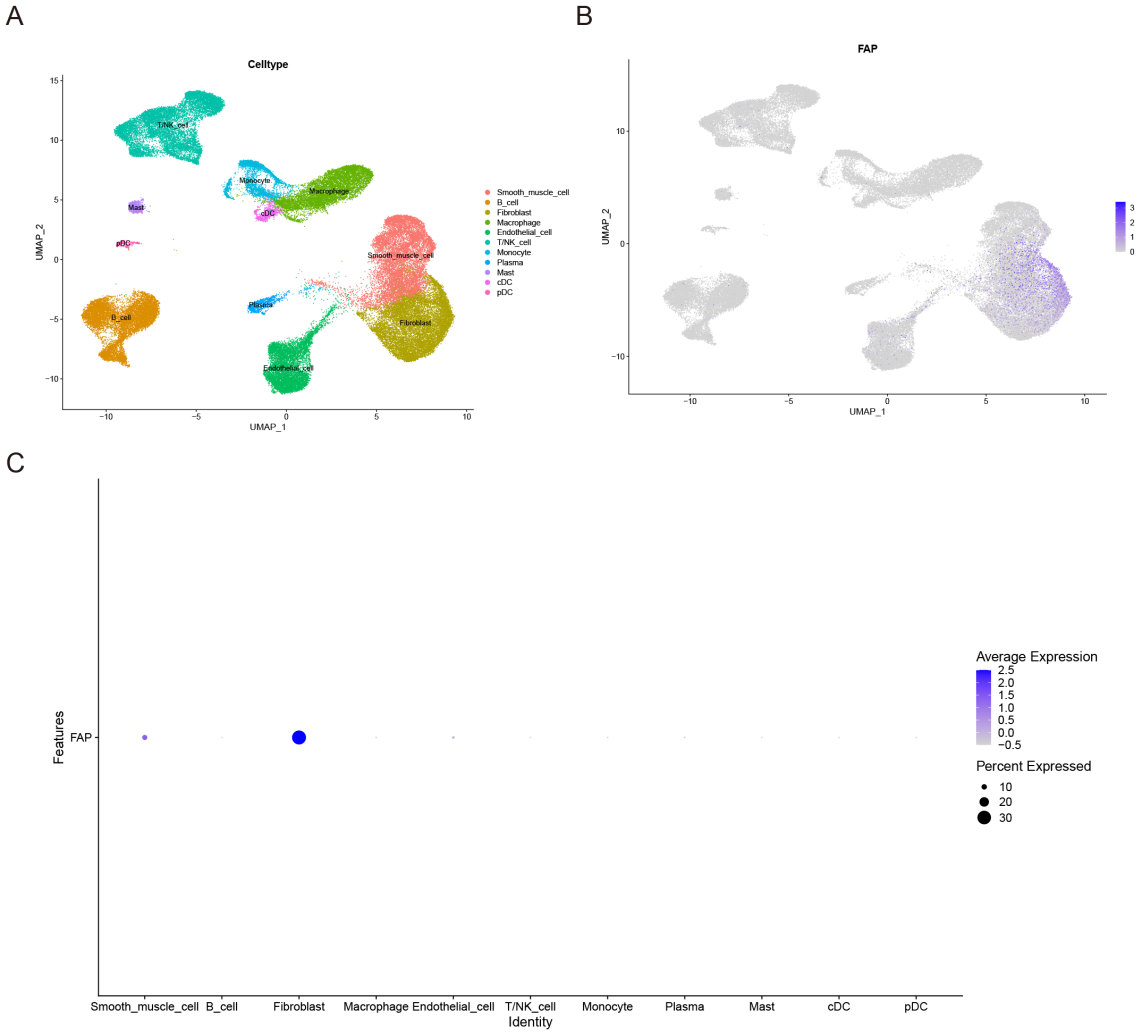
** Expression patterns of FAP in AAA and control samples.** (A) UMAP plot of 65031 cells from AAA and normal tissues. (B) Feature plot highlighting the spatial expression of FAP across cell clusters. (C) Dot plot showing the expression patterns of FAP in various cell clusters.

To further validate the cell type-specific expression patterns of these genes, we performed single-cell transcriptome analysis. Through integration of three scRNA-seq datasets using fastMNN batch correction, we analyzed 65,031 high-quality cells. UMAP clustering identified 11 distinct cell populations: smooth muscle cell, B cell, plasma cell, conventional dendritic cell (cDC), plasmacytoid dendritic cell (pDC), mast cell, macrophages, endothelial cell, natural killer (NK) cells, fibroblasts, monocyte, and T/NK cell hybrid (Figure 2A). Notably, FAP exhibited significant expression specificity in VSMCs and fibroblasts (Figure 2B-C), while the remaining six candidate genes showed varying cell-type specificities (Supplementary Figure 1A-F). These results suggest that the high expression of FAP may play a crucial role in the pathogenesis of AAA, as VSMCs are the predominant cell type of the aorta 16. Therefore, we further explored the role and mechanisms of FAP in AAA.

### FAP expression in human samples

**Figure 3:**
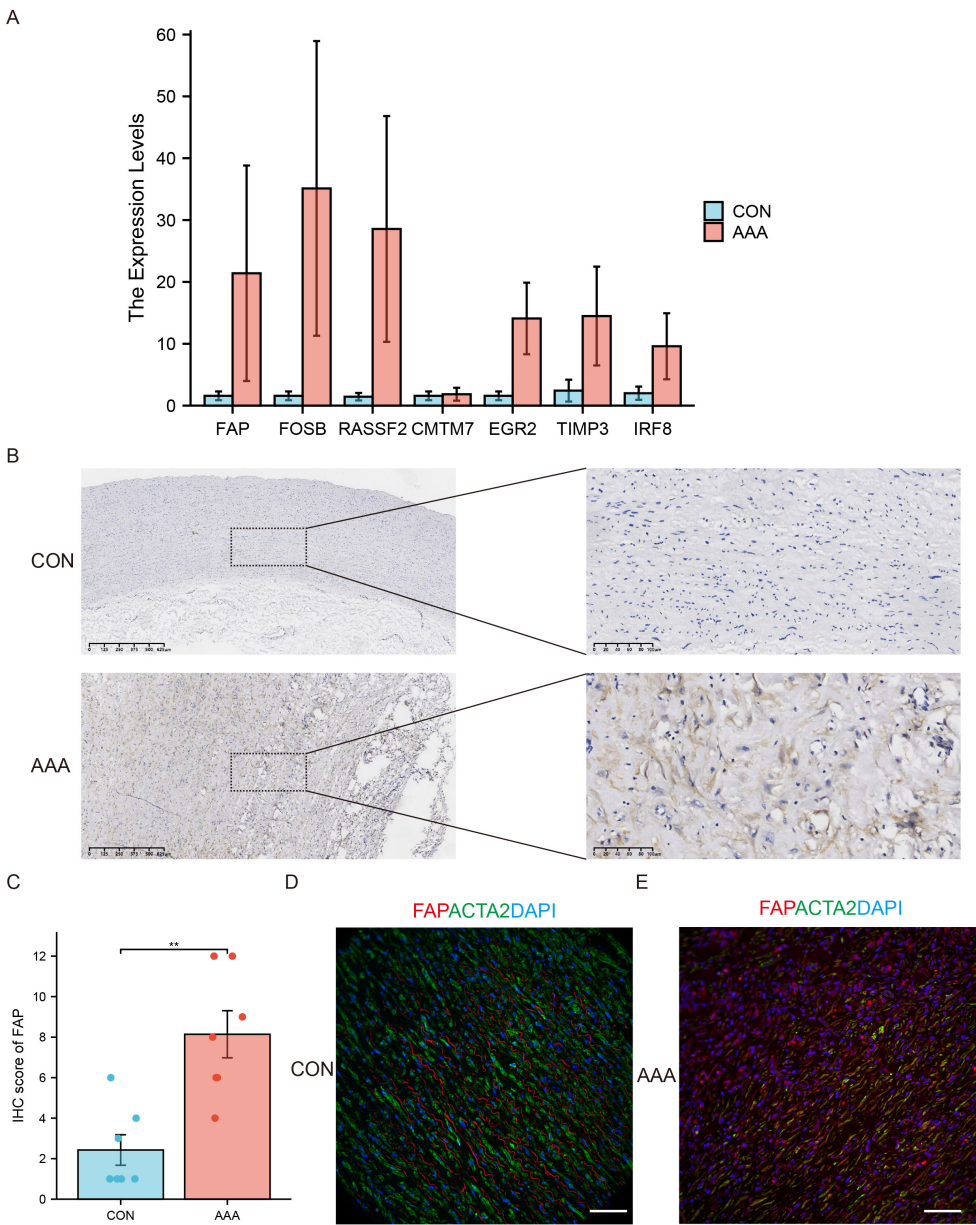
** Experimental validation of candidate genes.** (A) Expression patterns of seven candidate genes in AAA (N=6) and control tissues (N=5) by using qRT-PCR. (B) Immunohistochemistry (IHC) staining of FAP in human AAA samples (N=7) compared to normal tissues (N=7). (C) Quantitative analysis of FAP expression by IHC in AAA (N=7) and normal (N=7) tissues. (D) Immunofluorescence (IF) staining of AAA and normal tissues with DAPI (blue), FAP (red), and α-SMA (green), demonstrating partial colocalization of FAP with VSMCs (N=5). Scale bar: 50 µm. SVM, support vector machine; FAP, fibroblast activation protein; CON, healthy control; AAA, abdominal aortic aneurysm. *, P < 0.05; **, P < 0.01; ***, P < 0.001.

We performed RT-qPCR validation in AAA (N=6) and control (N=5) tissues (Figure 3A). Consistent with bioinformatics predictions, FAP, FOSB, and RASSF2 showed significant overexpression in AAA samples. While FOSB and RASSF2 have established roles in AAA literature 17, 18, our study represents the first report implicating FAP in aneurysm pathology. Therefore, we prioritized FAP as the primary research focus.

Immunohistochemical analysis of human AAA specimens versus controls confirmed elevated FAP protein levels in diseased tissues (Figure 3B-C). Immunofluorescence co-staining revealed partial colocalization of FAP with α-SMA-positive VSMCs, though overall FAP signal intensity was increased in AAA tissues (Figure 3D), suggesting potential post-translational regulation or cellular redistribution during disease progression.

### FAP Expression Associates with Macrophage Infiltration in AAA

**Figure 4:**
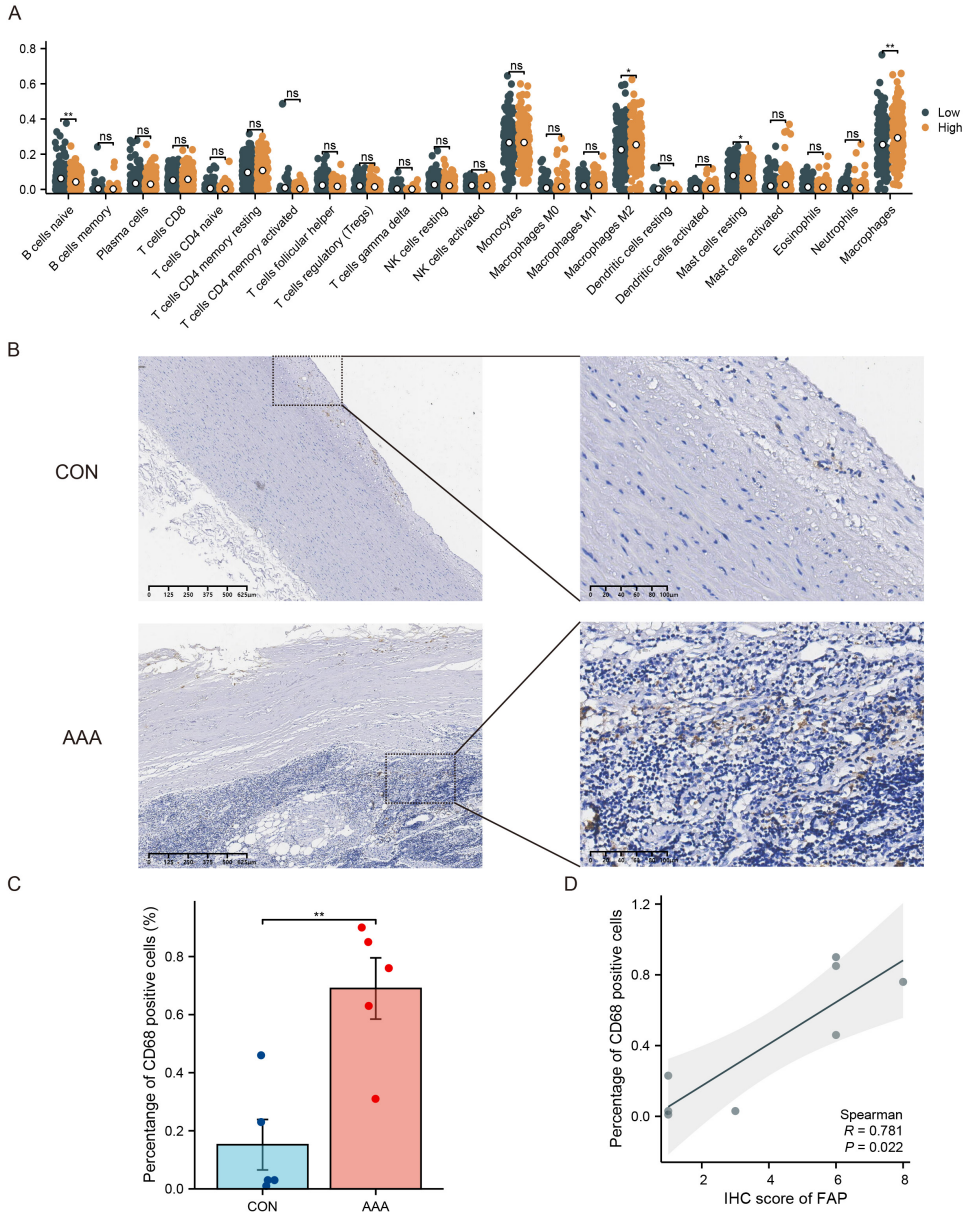
** FAP's role in immune cell infiltration in AAA.** (A) The proportion of 22 kinds of immune cells in different samples visualized from the boxplot. (B-C) IHC assessment of CD68+ cells in control and AAA tissue specimens. CD68 expressions were elevated. (D) Correlations of tissues levels of FAP expression with CD68 cells percentage. CON, healthy control; AAA, abdominal aortic aneurysm; *, P < 0.05; **, P < 0.01; ***, P < 0.001.

Immune cell infiltration is important in AAA, and it is unclear whether FAP is involved in immune cell infiltration. Immune cell infiltration in AAA was evaluated using bulk RNA-seq data. Based on the expression levels of FAP in VSMCs, AAA samples were categorized into FAP-high and FAP-low groups. CIBERSORT analysis revealed a significant increase in macrophage infiltration within the aneurysmal wall in the FAP-high AAA group (Figure 4A).

The role of VSMCs in aortic aneurysm pathogenesis has been well-established 16. We hypothesized that crosstalk between different VSMCs subpopulations could significantly contribute to disease progression. Based on those results, we speculate that FAP is associated with macrophage infiltration. Significantly, tissues from AAA patients displayed an elevated proportion of CD68-positive cells (p < 0.05) (Figure 4B-C), which led us to focus specifically on macrophage infiltration in AAA pathogenesis. Immunohistochemical analysis of consecutive tissue sections demonstrated a strong correlation between FAP expression and macrophage accumulation within the aneurysmal aortic wall (Figure 4D).

### FAP inhibition attenuates PPE-induced AAA formation in mice

**Figure 5:**
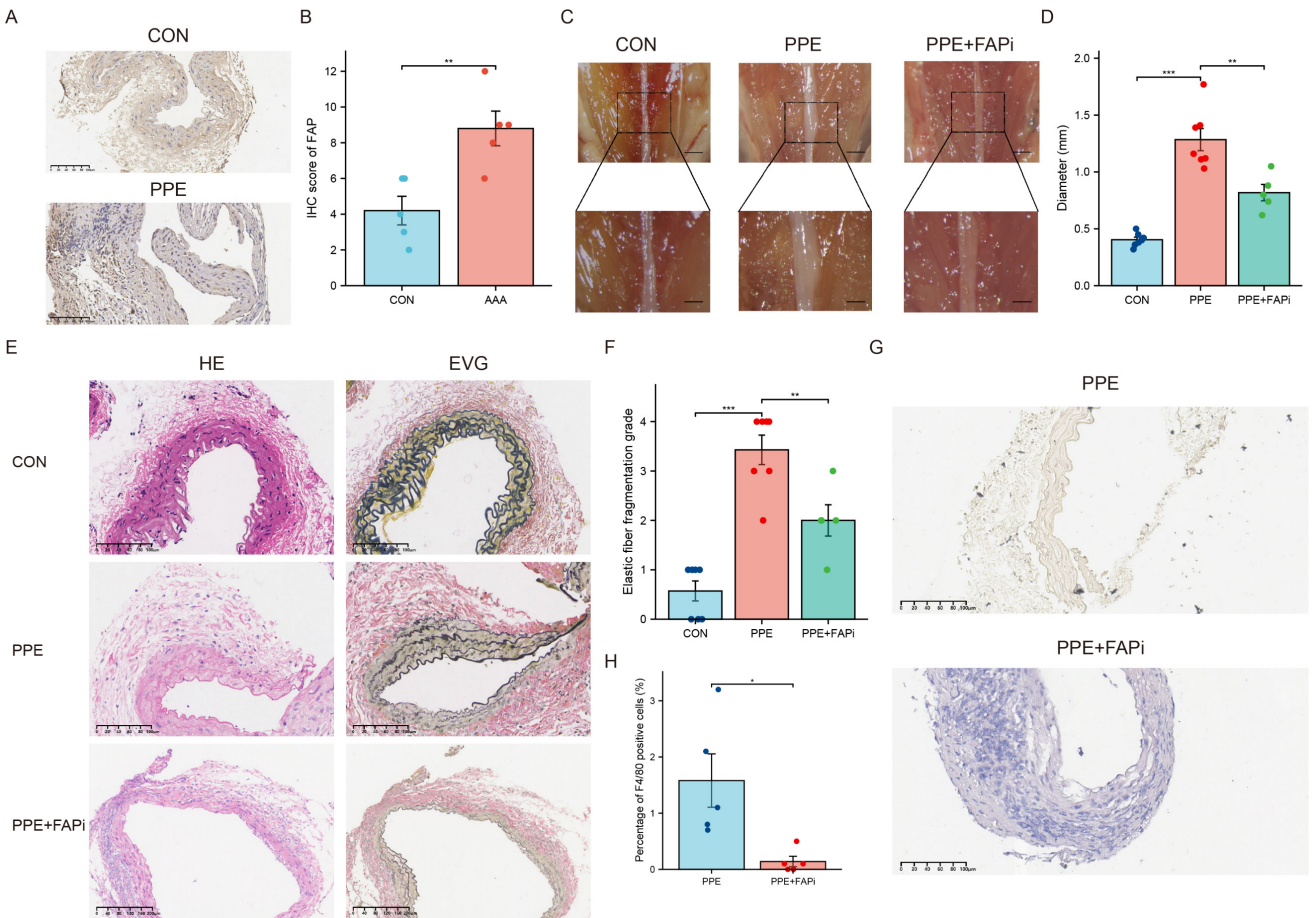
** Construction of the mouse AAA model.** (A) IHC of FAP in mouse AAA and normal tissues, N = 7. (B) Quantification of the expression of FAP. Data were analyzed using student t-test. (C) The morphogram of normal subrenal aortic artery and AAA. N = 6-10, Scale bar, 1 mm for upper panel and 0.5 mm for inferior panel. (D) Quantification of the maximum external diameter of abdominal aorta. Data were analyzed using student t-test. (E) The H&E and EVG staining images of normal subrenal aortic artery and AAA. N = 6-8. The red arrows indicate the location of breakages of elastic fibers. (F) The histograms of the elastic fiber fragmentation grade. Data were analyzed by one-way ANOVA. (G) IHC of F4/80 in mouse AAA and normal tissues, N = 5. (H) Quantification of the percentage of F4/80+ cells. Data were analyzed using student t-test. CON, DMSO control; PPE, porcine pancreatic elastase; FAPi, fibroblast activation protein inhibitor; EVG, Elastin Van Gieson; ns, no significance; *, P < 0.05; **, P < 0.01; ***, P < 0.001; ****, P < 0.0001.

AAA was induced in mice through PPE exposure. Two weeks after the treatment, IHC analysis demonstrated increased FAP expression in mouse AAA tissues (Figure 5A and 5B). Meanwhile, we used the FAPi Ac-Gly-BoroPro to investigate the impact of FAP on AAA. The results showed that FAPi-treated mice had a reduced AAA size compared to the control group (P <0.01) (Figure 5C-D). Histological analysis revealed clear signs of aortic lumen dilation and elastin degradation in the control group after AAA induction (Figure 5E-F). Notably, FAPi treatment partially mitigated these changes (Figure 5E-F). Moreover, inhibition of FAP could effectively reduce the F4/80+ cells accumulation (Figure 5G-H). These results suggest that FAPi may help limit the progression of AAA in mice.

### PDGF-BB increases FAP expression in VSMCs via EGR1 in VSMCs

**Figure 6:**
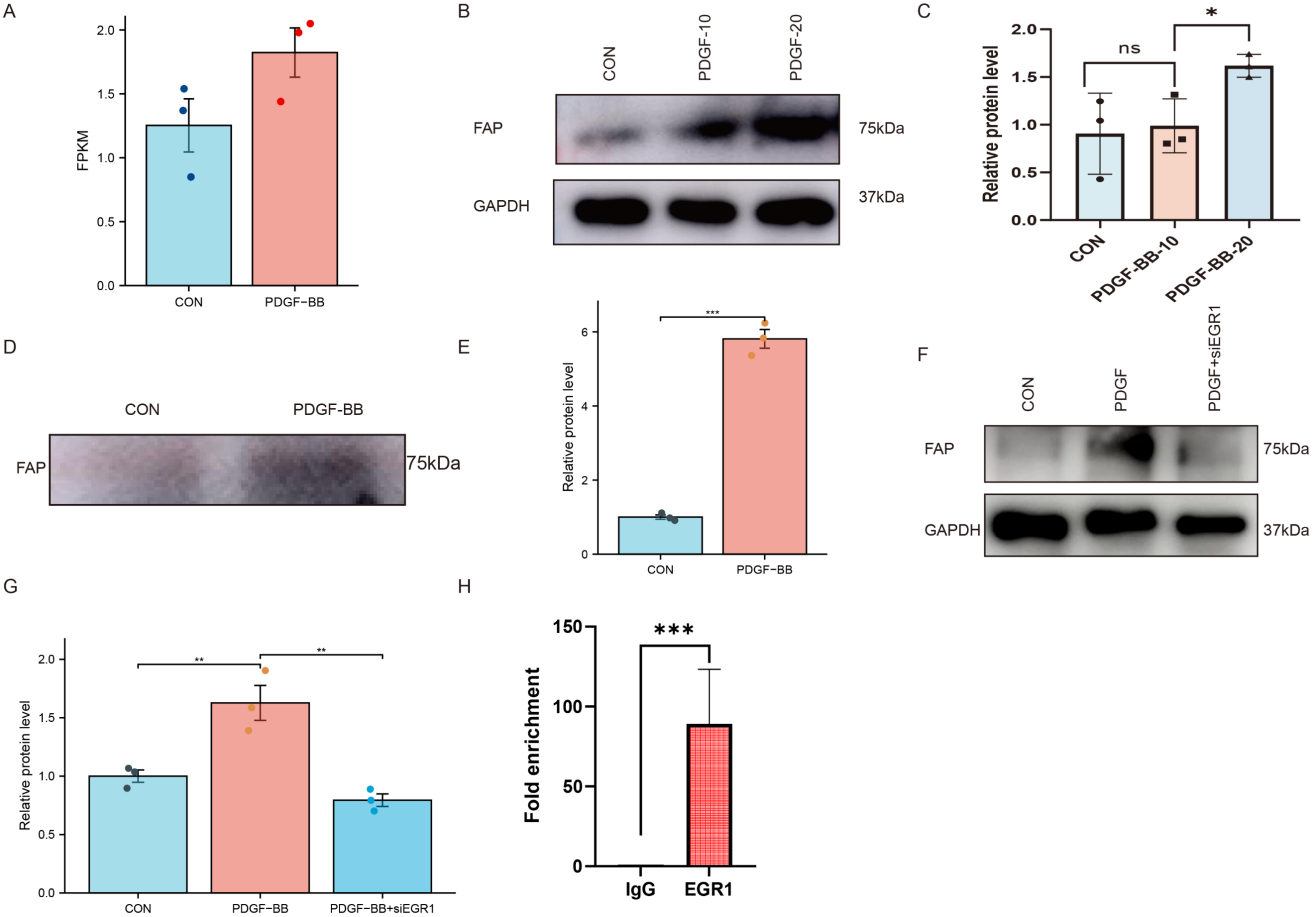
** PDGF-BB induces FAP expression in VSMCs via EGR1-mediated transcriptional activation.** (A) Scatter plot from bioinformatics analysis showing a positive correlation between PDGF-BB and FAP expression (FPKM values). (B-C) Western blot analysis of FAP protein expression in VSMCs treated with PDGF-BB (0,10, and 20 ng/ml, 24 hours), N = 3. Data were analyzed using Mann-Whitney U test. (D-E) Western blot analysis of FAP protein expression in VSMCs supernatant treated with PDGF-BB (0 or 20 ng/ml, 24 hours), N = 3. Data were analyzed using Mann-Whitney U test. (F-G) Western blot analysis of FAP expression in VSMCs with or without silencing EGR1 and PDGF-BB induction, N = 3. Data were analyzed using student t-test. (H) Statistical quantification of EGR1 binding to the FAP promoter, N = 3. Data were analyzed using student t-test. Data are presented as mean ± SEM. FAP, fibroblast activation protein; Con, healthy control; AAA, abdominal aortic aneurysm. *, P < 0.05; **, P < 0.01; ***, P < 0.001; ****, P < 0.0001.

Given the PDGF-BB upregulation in AAA tissues which may promote the expression of FAP 19, we initially examined whether PDGF-BB could induce FAP expression in VSMCs. We analyzed the GSE241545 dataset and found that PDGF-BB may promote FAP expression, although no statistically significant differences were observed (Figure 6A). We hypothesize that PDGF-BB can modulate FAP expression in VSMCs. Western blotting showed FAP protein levels, normalized to GAPDH, increased with PDGF-BB stimulation (0, 10, 20 ng/ml, 24h) (Figure 6B-C). Since FAP can be secreted into the extracellular space, we measured the expression of FAP in the cell supernatant. Given the potential of PDGF-BB to promote cell proliferation, we seeded VSMCs at the same cell density in the culture medium. We observed that stimulation of VSMCs with PDGF-BB (20 ng/ml, 0-24 h) significantly upregulated FAP expression in the cell supernatant (Figure 6D-E). These results demonstrated the pivotal role of PDGF-BB in increasing FAP expression in VSMCs.

By querying the TRUST database (https://www.grnpedia.org/), we identified EGR1 as the only transcription factor of FAP. Previous studies have reported that PDGF-BB can enhance the expression of EGR1 which leading us to propose that EGR1 may mediate the regulation of FAP by PDGF-BB. To further investigate, we aimed to determine if EGR1 silencing could inhibit PDGF-BB-mediated FAP upregulation. Transfection of VSMCs with EGR1 siRNA successfully blocked the induction of FAP expression by PDGF-BB (20 ng/ml, 24 hours) (Figure 6F and G). Taken together, these results suggest PDGF-BB can increase FAP expression levels in VSMCs via EGR1.

### EGR1 binding to FAP promoter in VSMCs

The FAP promoter regions containing transcription factor binding sites are critical for PDGF-BB-induced FAP expression. To confirm this speculation, chromatin immunoprecipitation (ChIP) studies coupled with quantitative real-time PCR were used to amplify the FAP promoter binding site following immunoprecipitation with EGR1 in VSMCs. To validate chromatin fragmentation efficiency prior to immunoprecipitation, DNA gel electrophoresis confirmed successful ultrasonic shearing of chromatin into fragments before immunoprecipitation (Supplementary Figure 2). Subsequent ChIP-qPCR analysis demonstrated significant enrichment of EGR1 binding to the FAP promoter compared to IgG controls (Figure 6H).

### Vessel wall FAPi uptake

**Figure 7:**
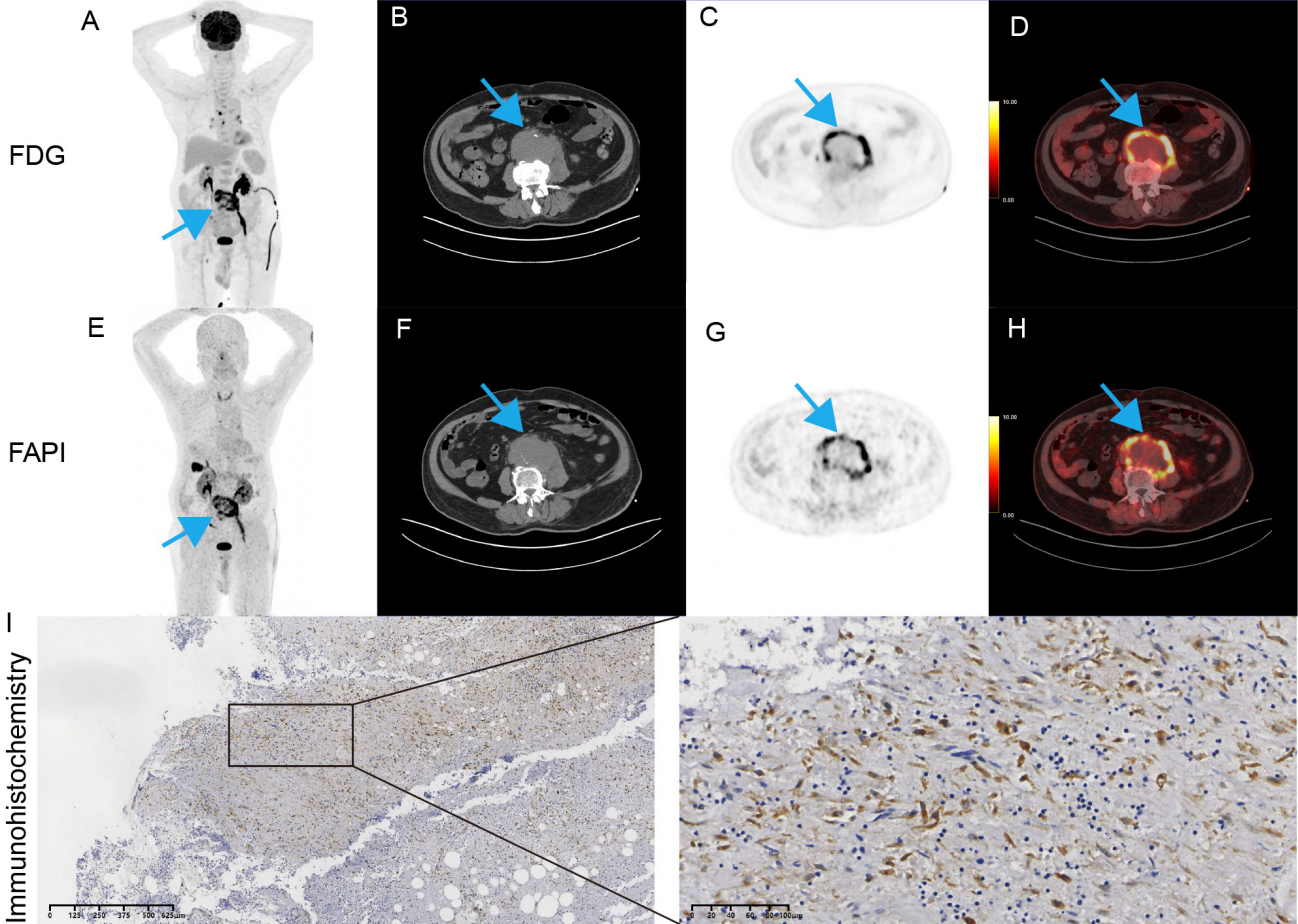
** PET/CT and pathology images of our patient.** (A) MIP. (B) axial CT. (C) axial PET. (D) fused PET/CT. (E) MIP. (F) axial CT. (G) axial PET. (H) fused PET/CT. (I) Representative images of FAP in postoperative tissues by immunohistochemistry.

Imaging based on FAPi has been widely used in clinical practice 20. Given that FAP is associated with inflammation and infected AAA are characterized by acute inflammatory infiltration 21, 22, we first explored the potential value of FAP in patients with suspected infectious AAA. A 90-year-old male patient presented to the emergency department with fever and abdominal pain. He had experienced intermittent fever for the past week. Laboratory tests showed elevated white blood cell count (15.62 × 10^9/L) and markedly increased procalcitonin (10.4 ng/mL), indicating systemic infection. To further evaluate the inflammatory activity of the aneurysm, an ^18^F-FDG PET/CT scan was finished according to recommendation 23. This scan demonstrated abnormal uptake in the abdominal aortic wall with SUVmax of 15.7 and a TBR of 6.0 (Figure 7A-D). Given the patient's history of diabetes, which could interfere with glucose metabolism, the clinical team decided to perform an ^18^F-FAPi PET/CT scan free of charge to explore potential application value. The ^18^F-FAPi PET/CT revealed similar abnormal uptake in the aortic wall, with a slightly higher SUVmax of 16.8 and a TBR of 5.6 (Figure 7E-H). These findings were comparable to the ^18^F-FDG PET/CT results, suggesting active infection and inflammation within the aneurysm wall. The patient underwent open surgical repair of the aneurysm. Immunohistochemical staining showed strong expression of FAP within the aneurysm wall (Figure 7I).

## Discussion

AAA imposes significant clinical and economic burdens due to the lack of effective therapies. Therefore, identifying therapeutic targets for AAA is crucial. *In vivo* studies, we used a PPE-induced AAA mouse model demonstrated that FAP inhibition reduced aneurysm progression and macrophage infiltration, further supporting its role in AAA pathogenesis. Meanwhile, PDGF-BB promotes the transcription of EGR1, thereby increasing FAP expression. These results explain why FAP is highly expressed in AAA and how it contributes to the progression of abdominal aortic aneurysm.

Research has demonstrated that fibroblast activation protein (FAP), a type-II transmembrane serine protease, exhibits minimal expression in healthy tissues but is significantly upregulated in various pathological conditions such as fibrosis, arthritis, and malignancies 24. Notably, genetic ablation of FAP has been shown to attenuate the development of experimental atherosclerosis, enhance plaque stability, and reduce collagen degradation 25. However, the changes and role of FAP in AAA have not been reported. By analyzing bulk and single-cell data, we found that FAP is highly expressed in VSMCs, which was further confirmed by immunohistochemistry and immunofluorescence. These findings indicate that FAP may significantly contribute to the initiation and progression of AAA.

Next, we investigated the impact of FAP on AAA. After injection a FAP-specific inhibitor to the PPE-induced mouse AAA model, we observed a reduction in aneurysm size, demonstrating that FAP can promote aneurysm formation. Furthermore, immune infiltration analysis, cell communication analysis, and immunohistochemical analysis suggest that FAP may promote AAA by influencing macrophage infiltration. Despite the absence of FAP expression in macrophages, a significant association was observed between FAP levels and the extent of macrophage infiltration within human aortic plaques 26. Another possible mechanism is that FAP selectively cleaves type I collagen, leading to increased macrophage adhesion, which in turn promotes tumor progression 27. However, they did not investigate the intracellular mechanisms by which FAP promotes the increase of macrophages. There is a lack of further investigation into its role in macrophage function such as polarization in inflammatory diseases. Further investigation into the modulation of FAP expression and its effects on macrophage function could provide novel strategies for controlling tissue remodeling and inflammatory responses in AAA and other cardiovascular pathologies.

Moreover, we investigated how FAP expression is upregulated in VSMCs. A study revealed elevated FAP expression in VSMCs within thin-cap atheromas of human aortic biopsies. This upregulation was driven by TNFα secreted from macrophages, and FAP levels showed a positive correlation with the extent of macrophage infiltration 26. Additionally, it is plausible that PDGF may serve as another activator of FAP expression, although conclusive evidence supporting this hypothesis remains unpublished 28. As is well known, PDGF-BB is enriched in AAA 19, 29. In addition to its diverse downstream functions, previous research has also extensively explored the regulation of FAP expression at both transcriptional and protein levels., involving factors such as TGF-β, anticancer cytokines like interferon 1 (IFN1), miRNAs, and others 30. It has been implicated that EGR1 can boost FAP production 31. Furthermore, our research demonstrates that PDGF-BB enhances FAP expression in VSMCs by activating the FAP promoter, with EGR1 playing a crucial role in this process, highlighting the importance of the EGR1/FAP axis in PDGF-BB-mediated cellular responses. Building on current findings, we provide valuable insights into PDGF-BB's role in vascular diseases such as AAA.

Quinoline-derived FAPi have recently gained significant attention in nuclear medicine worldwide, owing to their potential in cancer theranostics and the detection of diverse non-oncological disorders 32. Among these, ^18^F-FAPi stands out as a viable option for imaging malignancies and inflammatory cardiovascular conditions 30, 33. The broader adoption of PET/CT in clinical practice holds promise for enhancing the diagnosis and management of AAA patients 34, although the specific utility of FAPi PET in AAA remains to be thoroughly investigated. In summary, FAPi PET enables the detection of cardiovascular diseases such as tissue fibrosis and arterial inflammation by visualizing fibroblast activation protein 11. Notably, FAPi demonstrates significantly lower physiological accumulation in the mediastinal region, particularly in the myocardium and blood pool, compared to ¹⁸F-FDG 35. This favorable biodistribution suggests that FAPi PET may offer superior diagnostic value over ¹⁸F-FDG PET for cardiovascular applications. In our research, an infective aortic aneurysm exhibited abnormal ^18^F-FDG uptake, corroborated by ^18^F-FAPi PET/CT, highlighting that the advancement of FAPi for PET/CT has marked a significant milestone in molecular imaging. However, several variables remain unresolved—including the optimal FAPi tracer selection, appropriate acquisition protocols, and reliable qualitative/semi-quantitative uptake metrics—which currently limit the utility of FAPi PET for evaluating inflammatory fibrotic processes 36. Nevertheless, existing evidence is largely based on retrospective studies, and data for many potential applications remain limited. To integrate FAPi imaging into clinical guidelines and fully realize its capabilities, well-structured patient cohorts and, ideally, prospective randomized trials are essential.

However, this study primarily focused on *in vitro* experiments and did not explore the potential application of nuclear therapy in treating AAA, which could offer a promising therapeutic approach. In addition, we primarily use the PPE-induced mouse AAA model to validate FAP function, but this model may not fully replicate human AAA pathology.

While our case study demonstrates promising FAPi tracer uptake in an infectious AAA, several limitations must be acknowledged. First, the FAPi imaging findings from this single infectious AAA case cannot be extrapolated to conventional non-infectious AAA, as the observed FAP expression patterns may represent pathogen-specific inflammatory responses rather than general AAA pathophysiology. Notably, FAPI imaging in non-infectious AAA remains investigational, though prior studies support its broader potential. For instance, two clinical reports validated FAPi uptake in aortic dissection and ascending aortic aneurysm 37, 38, as well as rabbit aneurysm models 39, confirmed its feasibility for non-infectious vascular imaging. In future research, we plan to incorporate nuclear therapy strategies and employ various animal models, including gene knockout mice, to further explore FAP's role in AAA and validate its therapeutic potential *in vivo*.

In conclusion, FAP expression is mediated by PDGF-BB through EGR1 in VSMCs. *In vivo* inhibition of FAP reduced aneurysm progression and macrophage infiltration, highlighting its therapeutic potential. Targeting FAP may offer a promising strategy to modulate inflammation and prevent AAA progression.

## Supplementary Material

Supplementary figures and tables.

## Figures and Tables

**Table 1:** The primer sequences used for real-time PCR

RNA	Primer
Human-FAP-RT-F	ATGAGCTTCCTCGTCCAATTCA
Human-FAP-RT-R	AGACCACCAGAGAGCATATTTTG
Human-FOSB-RT-F	GCTGCAAGATCCCCTACGAAG
Human-FOSB-RT-R	ACGAAGAAGTGTACGAAGGGTT
Human-RASSF2-RT-F	AAGAAGACGAGTTCATTGTGGAG
Human-RASSF2-RT-R	GAATGCGTTCGTTGTCATCCT
Human-CMTM7-RT-F	GCCCCTGTCGATCTTTGGTTT
Human-CMTM7-RT-R	TGGACTGGGTTACACACGAGA
Human-EGR1-RT-F	TCAACATTGACATGACTGGAGAG
Human-EGR1-RT-R	AGTGAAGGTCTGGTTTCTAGGT
Human-TIMP3-RT-F	CATGTGCAGTACATCCATACGG
Human-TIMP3-RT-R	CATCATAGACGCGACCTGTCA
Human-IRF8-RT-R	ATGTGTGACCGGAATGGTGG
Human-IRF8-RT-R	AGTCCTGGATACATGCTACTGTC
Human-GAPDH-RT-F	GGAGCGAGATCCCTCCAAAAT
Human-GAPDH-RT-R	GGCTGTTGTCATACTTCTCATGG

**Table 2:** The 22 upregulated and 2 downregulated genes

Gene	Direction
FOSB	Up
RASSF2	Up
GBP5	Up
CMTM7	Up
MARCKS	Up
FOS	Up
MX2	Up
CD74	Up
UCP2	Up
TMEM71	Up
EGR2	Up
TIMP3	Up
VCAM1	Up
LCP1	Up
PLCG2	Up
MFAP5	Up
IRF8	Up
CTSC	Up
STAT1	Up
CXCR4	Up
NUP210	Up
FAP	Up
NXPH3	Down
ACADL	Down
