# Supplementary material for: PDGF-BB/EGR1 Axis Drives Fibroblast Activation Protein Expression to Promote Abdominal Aortic Aneurysm
Source: Int J Med Sci. 2025 Jun 5;22(11):2816–29. doi: 10.7150/ijms.114429 (PMC12163607; doi:10.7150/ijms.114429)
Supplement: Supplementary file 1 — Supplementary figures and tables. [file ijmsv22p2816s1.pdf]

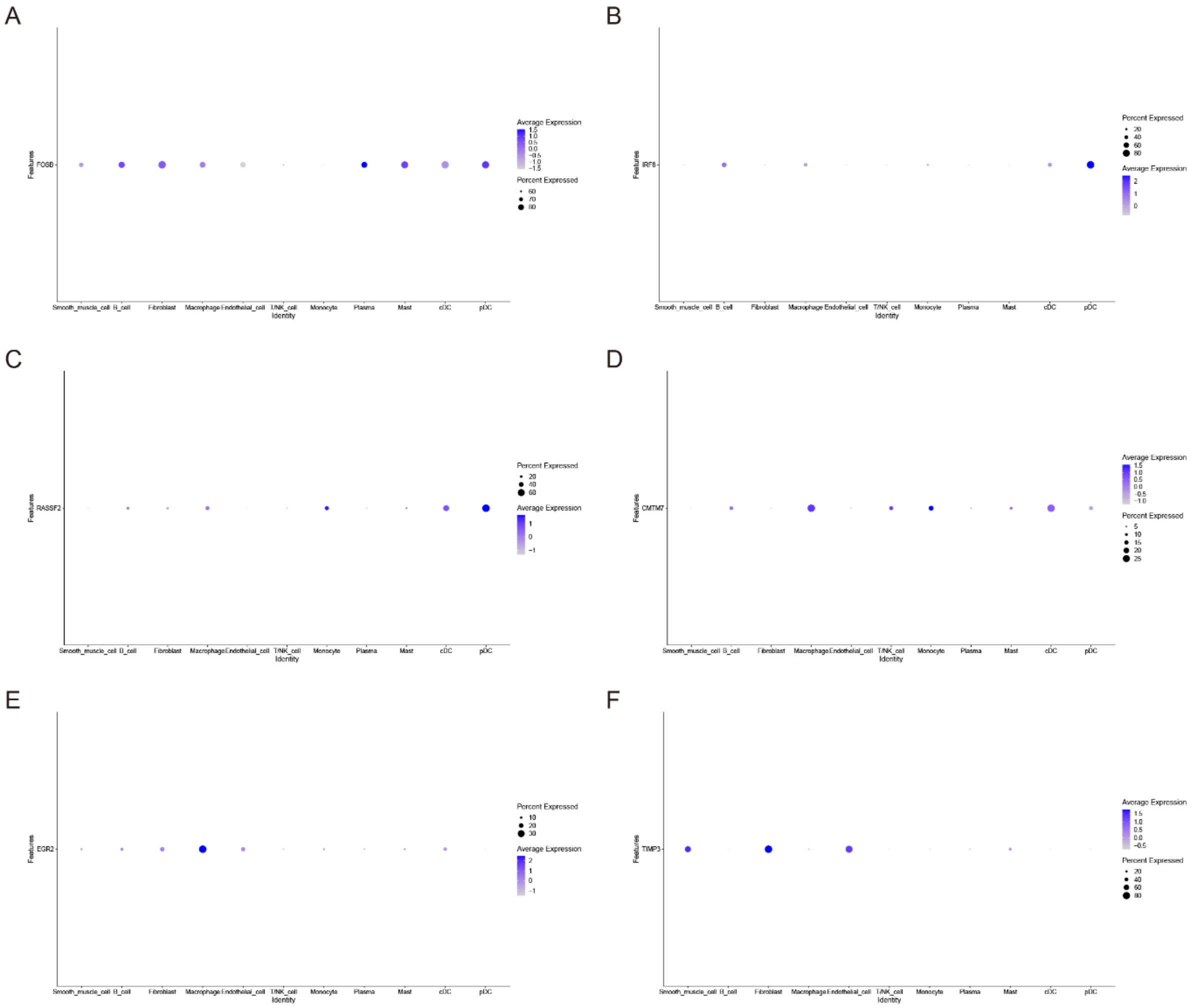

Supplementary Figure 1. (A-F) Dot plot showing the expression patterns of FOSB, IRF8, RASSF2, CMTM7, EGR2, and TIMP3 in various cell clusters.

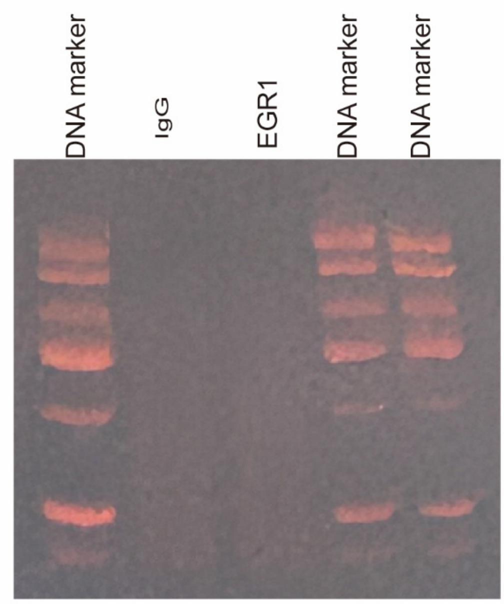

Agarose gel electrophoresis of DNA fragments after ultrasonic shearing before immunoprecipitation.

Supplementary Figure 2.
